# Case Report: Total hip arthroplasty via the direct anterior approach using a cementless revision stem without corrective osteotomy for fibrous dysplasia

**DOI:** 10.3389/fsurg.2026.1913902

**Published:** 2026-09-11

**Authors:** Tingjun Wang, Shiwei Liu, Yanlie Li, Jingming Zhang, Jiaying Wang, Wei Li, Guoliang Wang

**Affiliations:** Joint Surgery Department, Weifang People’s Hospital, Shandong Second Medical University, Weifang, China

**Keywords:** case report, direct anterior approach, fibrous dysplasia, shepherd's crook deformity, total hip arthroplasty

## Abstract

**Background:**

Fibrous dysplasia (FD) of the proximal femur frequently leads to secondary hip osteoarthritis and shepherd's crook deformity. Total hip arthroplasty (THA) in this setting typically requires corrective osteotomy and bone grafting. A simplified, osteotomy-free strategy has not been well characterised.

**Case presentation:**

A 50-year-old woman with monostotic FD and moderate shepherd's crook deformity (neck-shaft angle 110°) underwent primary THA via the direct anterior approach using a fully hydroxyapatite-coated cementless revision stem, without corrective osteotomy or bone grafting. Intraoperative blood loss was 200 mL. Full weight-bearing was achieved on postoperative day one. At six months, the Harris Hip Score improved from 64 preoperatively to 90, and radiographs confirmed stable implant fixation.

**Conclusions:**

In selected patients with monostotic FD, neck-shaft angle ≥110°, and adequate distal bone stock, this simplified approach permits immediate weight-bearing and excellent early function. These are strictly preliminary results from a single case, and the long-term safety and durability of this strategy remain unproven.

## Introduction

Fibrous dysplasia (FD) is a rare benign skeletal disorder first described by Lichtenstein in 1938, characterized by the replacement of normal lamellar bone and hematopoietic marrow by aberrant fibro-osseous tissue (1). It arises from a post-zygotic activating mutation in the GNAS gene, which encodes the alpha subunit of the stimulatory G protein (Gs*α*). This mutation drives constitutive activation of adenylyl cyclase and elevated intracellular cyclic adenosine monophosphate (cAMP), which in turn impairs osteoblastic differentiation and forms disorganized woven bone within a fibrous stroma (2). Accounting for 2.5% of all bone lesions and 7% of benign skeletal dysplasias (3), FD presents as either monostotic or polyostotic disease, with craniofacial bones and long bones as the predominantly affected sites (4). The proximal femur is a commonly affected site, where the mechanical weakness of dysplastic bone leads to progressive structural deformity. This deformity, in conjunction with limb length discrepancy and altered hip biomechanics, predisposes patients to secondary hip osteoarthritis. Consequently, it is estimated that approximately 12% of patients with FD develop symptomatic end-stage hip osteoarthritis requiring total hip arthroplasty (THA) in their lifetime (5).

THA in FD patients poses formidable technical challenges for orthopaedic surgeons. Inherently poor bone quality, marked by thin, fragile cortices and an intramedullary canal filled with abnormal fibro-osseous tissue, compromises reliable primary implant fixation (6). Furthermore, distorted proximal femoral anatomy complicates femoral canal preparation and markedly increases the risk of intraoperative periprosthetic fracture during broaching and stem implantation (7). These factors render standard primary THA implants potentially unsuitable for complex cases, necessitating meticulous preoperative planning and individualized implant selection to optimize outcomes.

This case adds a simplified strategy to the existing armamentarium: a fully hydroxyapatite-coated cementless revision stem implanted via the direct anterior approach (DAA), without corrective osteotomy or structural bone grafting, enabling immediate full weight-bearing in monostotic FD with moderate deformity. We provide the rationale for this paradigm and discuss considerations derived from our experience that may inform patient selection.

## Case presentation

### Patient information

A 50-year-old female farmer (height 160 cm, weight 65 kg, BMI 25.4 kg/m²) presented to our outpatient clinic with a 4-year history of progressive right hip pain. The pain was characterized as a persistent dull ache, with a visual analog scale (VAS) score of 3 at rest and 7 with weight-bearing activities. Symptoms were exacerbated by farm work, prolonged walking, and stair climbing, and were partially relieved by rest and oral non-steroidal anti-inflammatory drugs (NSAIDs). She had experienced progressive deterioration of pain and mobility limitation over the preceding 6 months.

### Clinical findings

On physical examination, the patient ambulated with an antalgic gait and demonstrated a positive Trendelenburg sign. Palpation elicited mild discomfort over the anterior joint line. Leg length evaluation demonstrated a true shortening of the right lower extremity by approximately 1.0 cm radiographically, with associated mild atrophy of the right thigh musculature (thigh circumference 2 cm less than the contralateral side measured 10 cm proximal to the patella). The range of motion of the right hip was moderately restricted in all planes: flexion 90°, extension 10°, abduction 15°, adduction 15°, internal rotation 5°, and external rotation 10°. The Thomas test and Patrick's (FABER) test were negative. Distal neurovascular status was intact, with palpable dorsalis pedis and posterior tibial pulses. Preoperative functional assessment using the Harris Hip Score (HHS) yielded a total score of 64 points (pain subscore 20/44, function subscore 34/47), indicating moderate-to-severe functional impairment.

### Diagnostic assessment

**Figure 1:**
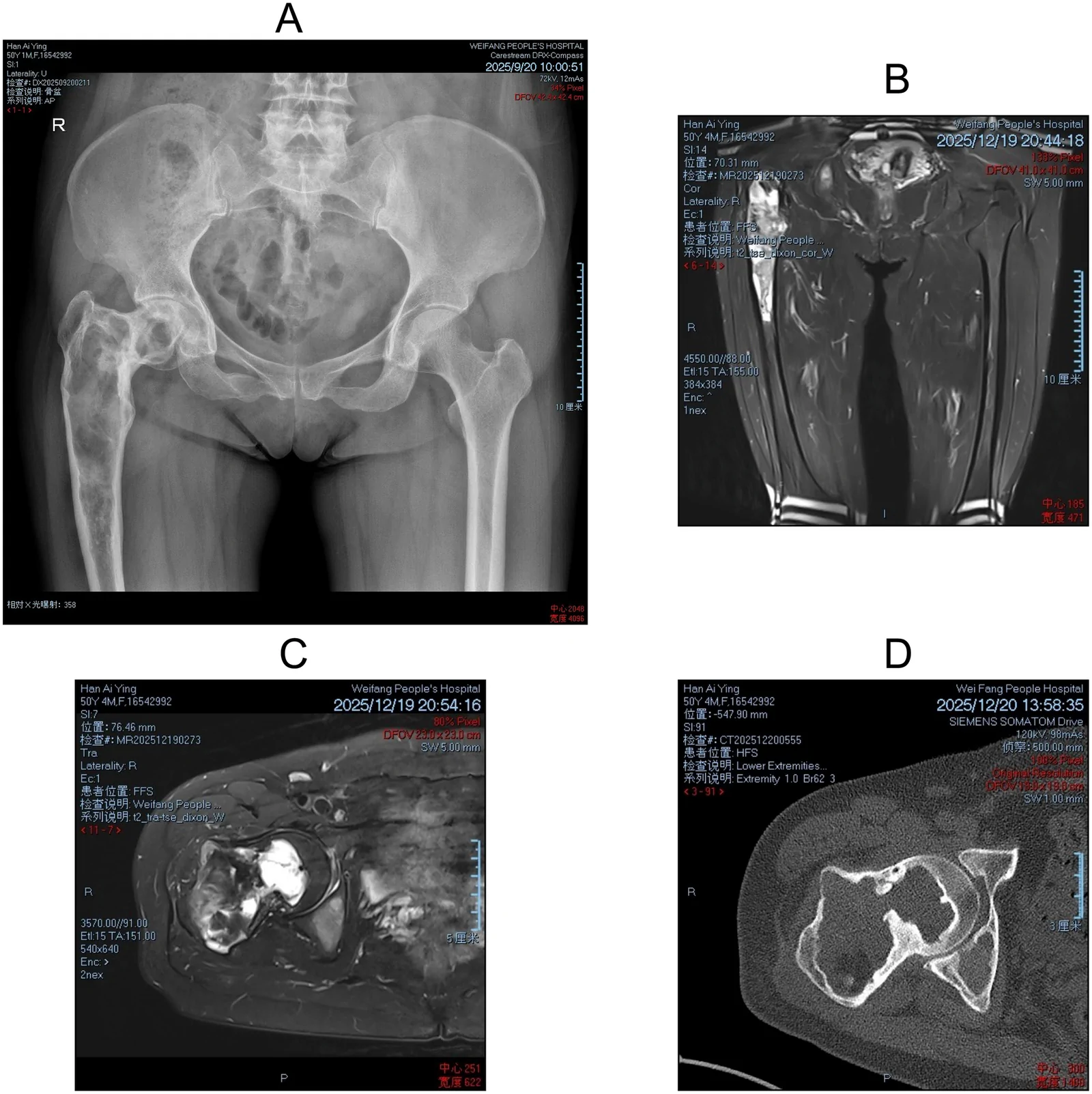
Preoperative imaging. **(A)** Anteroposterior radiograph of the hip. **(B)** Coronal MRI of the pelvis. **(C)** Axial MRI of the right proximal femur. **(D)** Axial CT scan of the right proximal femur.

Plain radiographs of the right hip revealed a characteristic “ground-glass” appearance of the proximal femur with a varus deformity of the femoral neck consistent with a classic shepherd's crook configuration (Figure 1A). The neck-shaft angle was reduced to approximately 110°, and the joint space was moderately narrowed with subchondral sclerosis. Magnetic resonance imaging (MRI) demonstrated an expansile lesion involving the right femoral head, neck, and proximal diaphysis, exhibiting heterogeneous signal intensity with areas of both T1 and T2 prolongation and heterogeneous hyperintensity on fat-suppressed sequences (Figures 1B,C). No soft tissue mass or cortical destruction was observed. Computed tomography (CT) further delineated the extent of bony involvement, confirming near-complete obliteration of the femoral medullary canal by fibro-osseous tissue (Figure 1D). The femoral cortices were markedly thinned (minimum thickness <2 mm). There was no evidence of pathologic fracture or features suggestive of malignant transformation. Notably, the diaphyseal bone below the isthmus appeared structurally intact with a cortical thickness >5 mm, suggesting adequate distal bone stock for diaphyseal fixation.

Endocrinological screening, including serum calcium, phosphate, parathyroid hormone, and thyroid function tests, was unremarkable. No clinical stigmata of McCune-Albright syndrome were identified. The diagnosis of monostotic FD was therefore established on the combined basis of clinical presentation, characteristic radiographic and histopathological findings, without targeted GNAS mutation testing.

### Preoperative planning and implant selection

Given the patient's age (50 years), functional demands as an active farmer, and the failure of conservative measures, a decision was made to proceed with total hip arthroplasty for end-stage secondary osteoarthritis. Preoperative templating was performed using standard digital radiographs. The critical technical challenges identified included: (1) severe varus deformity of the proximal femur (neck-shaft angle 110°), (2) near-complete obliteration of the medullary canal by dysplastic fibro-osseous tissue with a canal flare index <3.0, and (3) inherently poor proximal bone quality with cortical thickness <2 mm. These factors rendered a standard primary cementless stem, which relies on proximal metaphyseal fixation, potentially unsuitable due to inadequate rotational stability and an elevated risk of intraoperative fracture.

The decision to proceed without corrective osteotomy was based on the following preoperative assessment: (a) the neck-shaft angle was 110°, representing a moderate rather than severe varus deformity; (b) templating on anteroposterior and lateral radiographs confirmed a linear trajectory for a straight stem through the medullary canal without cortical impingement; (c) the diaphyseal bone distal to the lesion was of adequate quality, with a cortical thickness >5 mm on CT; and (d) the lesion was monostotic, without extensive cystic degeneration that would necessitate bone grafting. Based on this experience, we consider that the following factors may guide patient selection when contemplating this osteotomy-free approach: monostotic FD; neck-shaft angle ≥110°; absence of excessive anterior femoral bowing; adequate diaphyseal cortical bone stock (thickness >5 mm) distal to the lesion; and intraoperative fluoroscopic confirmation of unimpeded trial stem passage. These considerations are, however, derived from a single case and should not be regarded as validated selection criteria. Conversely, polyostotic FD with extensive proximal bone loss, neck-shaft angle <90°, severe anterior bowing precluding straight stem insertion, and large cystic defects requiring structural grafting likely warrant conventional osteotomy-based reconstruction.

After careful consideration, a fully hydroxyapatite-coated cementless revision stem (Corail Revision Stem; DePuy Synthes, Warsaw, IN, USA) was selected. This implant was chosen for its ability to achieve stable distal diaphyseal fixation, thereby bypassing the compromised proximal femoral segment. Its fully coated surface (150 μm hydroxyapatite), rectangular dual-tapered geometry, and extended length (150 mm) were anticipated to provide robust axial and rotational stability while bridging the dysplastic region. The rectangular cross-section is specifically advantageous, as it provides rotational control through vertical engagement at four cortical contact points along the diaphyseal isthmus, rather than relying on proximal metaphyseal press-fit. This is a mechanism that minimizes hoop stresses in the thin, fragile cortex characteristic of FD. Furthermore, the 150 μm HA coating has demonstrated the capacity to promote osseointegration even in the presence of residual fibrous tissue, a feature of particular relevance in FD where complete lesion debridement may not be achievable. In contrast to conical fluted stems that rely on metaphyseal engagement or cemented stems that depend on interdigitation with poor-quality cancellous bone, the revision stem offered the theoretical advantage of reliable distal fixation independent of proximal bone stock. Based on preoperative templating, acceptable component alignment could be achieved without a corrective femoral osteotomy, which was therefore deemed unnecessary.

The direct anterior approach was selected for its specific advantages in this complex case. Given the compromised abductor mechanism and posterior capsular stabilizers often observed in FD patients with proximal femoral deformity, an approach preserving these structures was considered paramount for postoperative stability and early mobilization. The DAA is performed through the intermuscular and internervous plane between the tensor fasciae latae and sartorius muscles, preserving the insertions of the gluteus medius and minimus as well as the integrity of the posterior capsule. This represents a distinct advantage over posterior or lateral approaches, which inherently require disruption of one of these critical stabilizing structures.

Following a thorough preoperative evaluation confirming the absence of contraindications to surgery, a detailed discussion was undertaken with the patient and her family, who expressed understanding of the proposed surgical plan and provided informed consent to proceed.

### Therapeutic intervention

Following the uneventful induction of general anesthesia, the patient was positioned in the standard lateral decubitus position and secured with anterior and posterior pelvic supports. A DAA was utilized, accessing the hip joint through the intermuscular interval between the tensor fasciae latae and the sartorius. Intraoperatively, identification of the intermuscular plane required careful dissection due to the distorted proximal femoral anatomy; the ascending branch of the lateral femoral circumflex artery was identified and ligated as a key landmark. The anterior capsule was incised and retracted, providing excellent visualization of the femoral neck despite the varus deformity.

**Figure 2:**
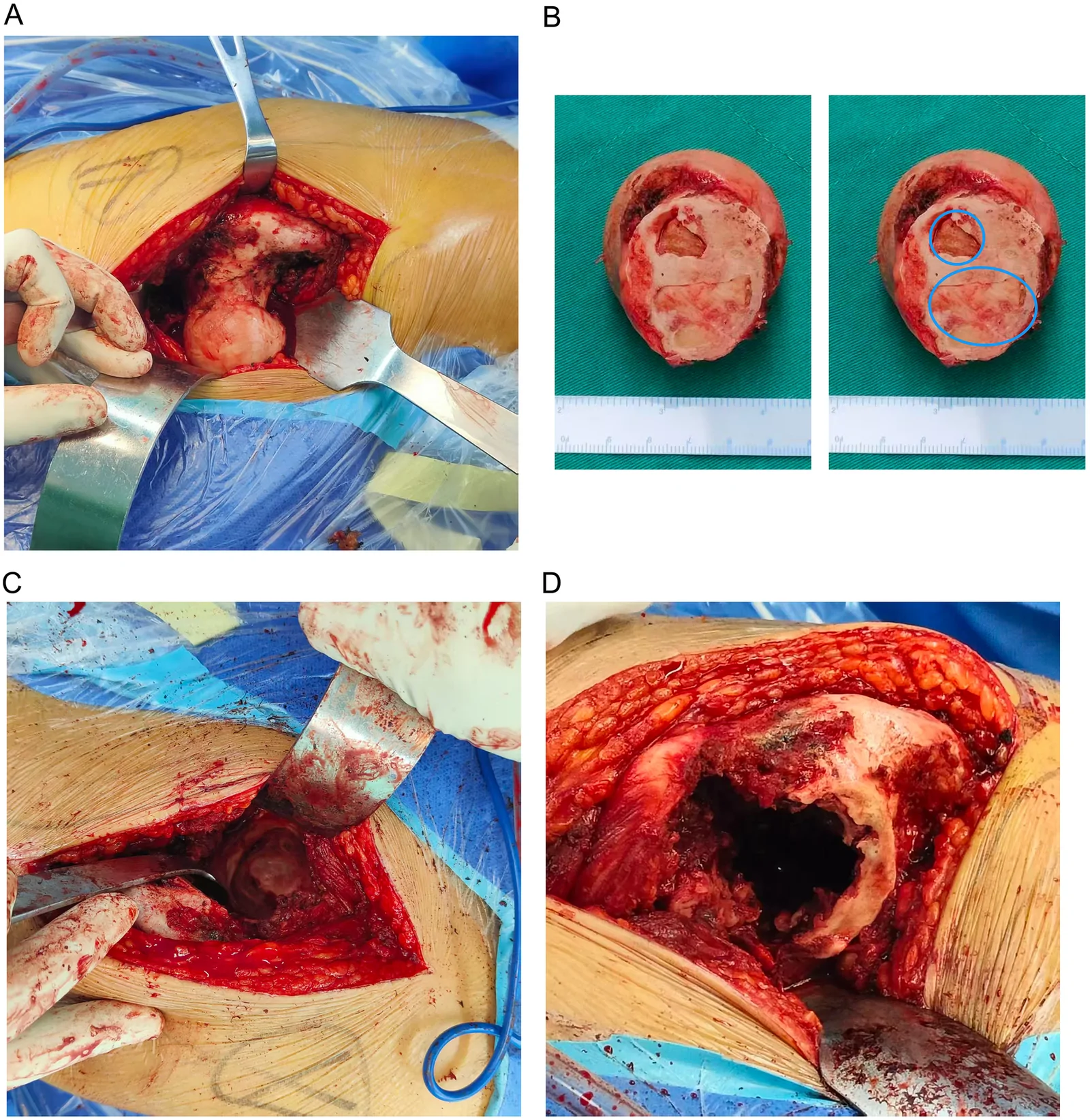
Intraoperative photographs and histopathological examination. **(A)** Intraoperative view after hip dislocation. **(B)** Excised femoral head; the blue circle indicates the firm, greyish-white “fish-flesh”-like fibro-osseous tissue. **(C)** Acetabulum after femoral neck osteotomy. **(D)** Calcar femorale after osteotomy.

Upon exposure of the hip joint, a moderate amount of clear, straw-colored synovial fluid was evacuated. The femoral head exhibited mild cartilage degeneration without significant collapse or flattening. The femoral neck was osteotomized 1.5 cm proximal to the lesser trochanter, and the femoral head was removed. Inspection of the osteotomized surface revealed that the medullary canal was completely filled and obliterated by firm, grayish-white, “fish-flesh”-like fibro-osseous tissue (Figures 2A–D). This tissue was meticulously curetted to facilitate canal preparation. Sequential reaming was performed with extreme care, beginning with an 8 mm reamer and gradually increasing in diameter under fluoroscopic guidance to avoid cortical perforation or fracture. Throughout reaming, intermittent fluoroscopy in both anteroposterior and lateral planes was employed to confirm central intramedullary positioning and to rule out cortical breach, given the obliteration of normal endosteal landmarks by FD tissue.

**Figure 3:**
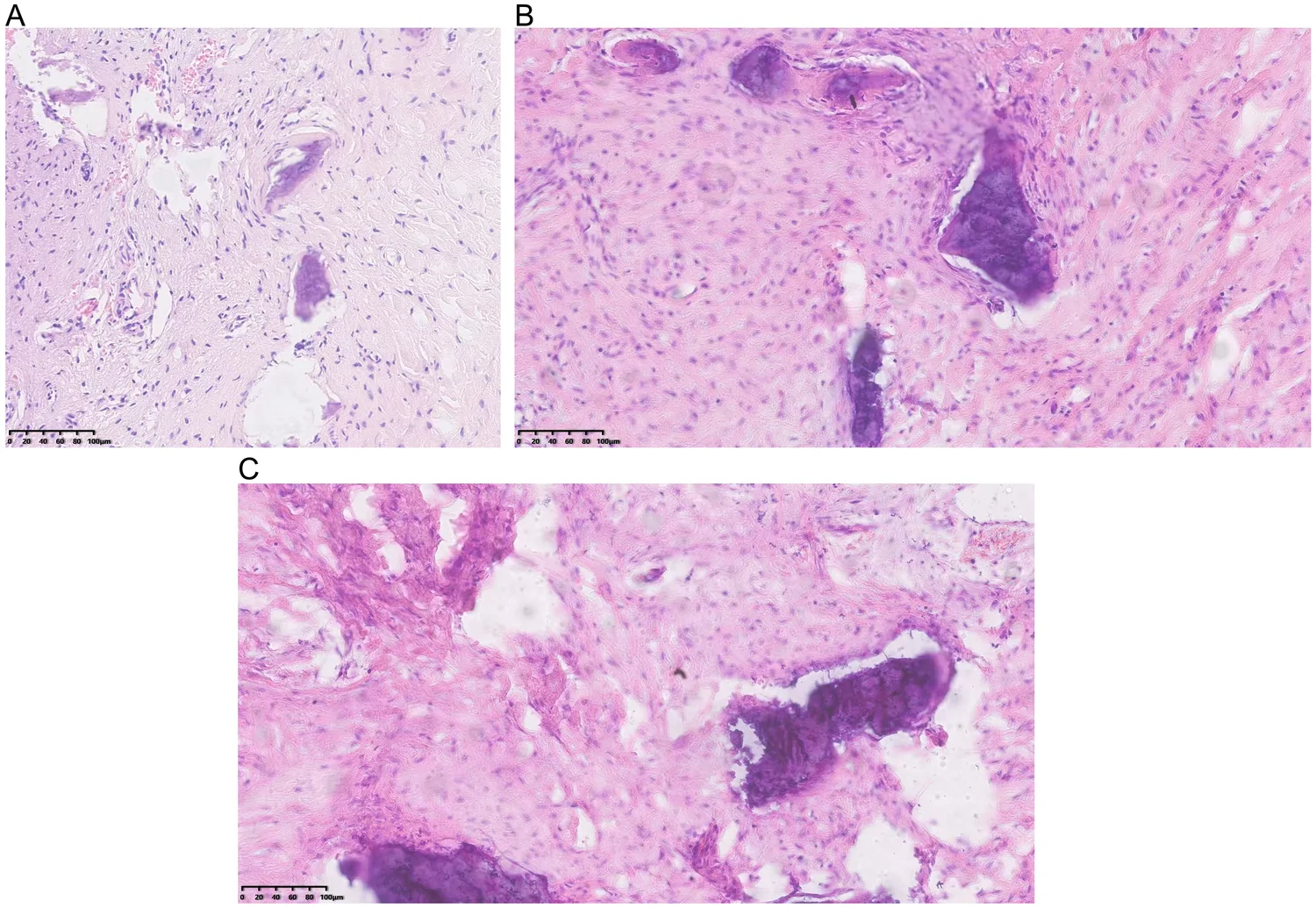
Histopathological examination. **(A–C)** Sections showing irregular woven bone trabeculae within a fibrous stroma, lacking osteoblastic rimming.

Ultimately, the canal was prepared to accept a size 15 implant. A 50 mm cementless acetabular component (POROCOAT; DePuy Synthes, USA) was implanted with two supplemental screws for enhanced fixation. A highly cross-linked polyethylene liner was inserted. A size 15 fully hydroxyapatite-coated Corail Revision Stem (REVISION 12/14 AMT; DePuy Synthes, USA) was impacted into the prepared canal. Excellent axial and rotational stability was confirmed by the absence of micromotion on torsional testing and a solid acoustic tone on impaction. A 32 mm +1 ceramic femoral head (Biolox delta; DePuy Synthes, USA) was then affixed to the trunnion, and the hip was reduced. Intraoperative fluoroscopy confirmed appropriate stem alignment and seating without evidence of cortical perforation or fracture. The hip was taken through a full range of motion, demonstrating stability in flexion, extension, and rotation without impingement. Intraoperative blood loss was approximately 200 mL. No intraoperative transfusion was required. Histopathological analysis revealed irregular, curvilinear trabeculae of woven bone lacking conspicuous osteoblastic rimming, dispersed within a moderately cellular fibrous stroma, consistent with FD (Figures 3A–C).

Venous thromboembolism (VTE) prophylaxis included intraoperative and postoperative intermittent pneumatic compression devices until full ambulation, and low-molecular-weight heparin (enoxaparin 4,000 IU subcutaneously once daily) initiated 6 h postoperatively and continued for 14 days.

### Postoperative course and early follow-up

**Figure 4:**
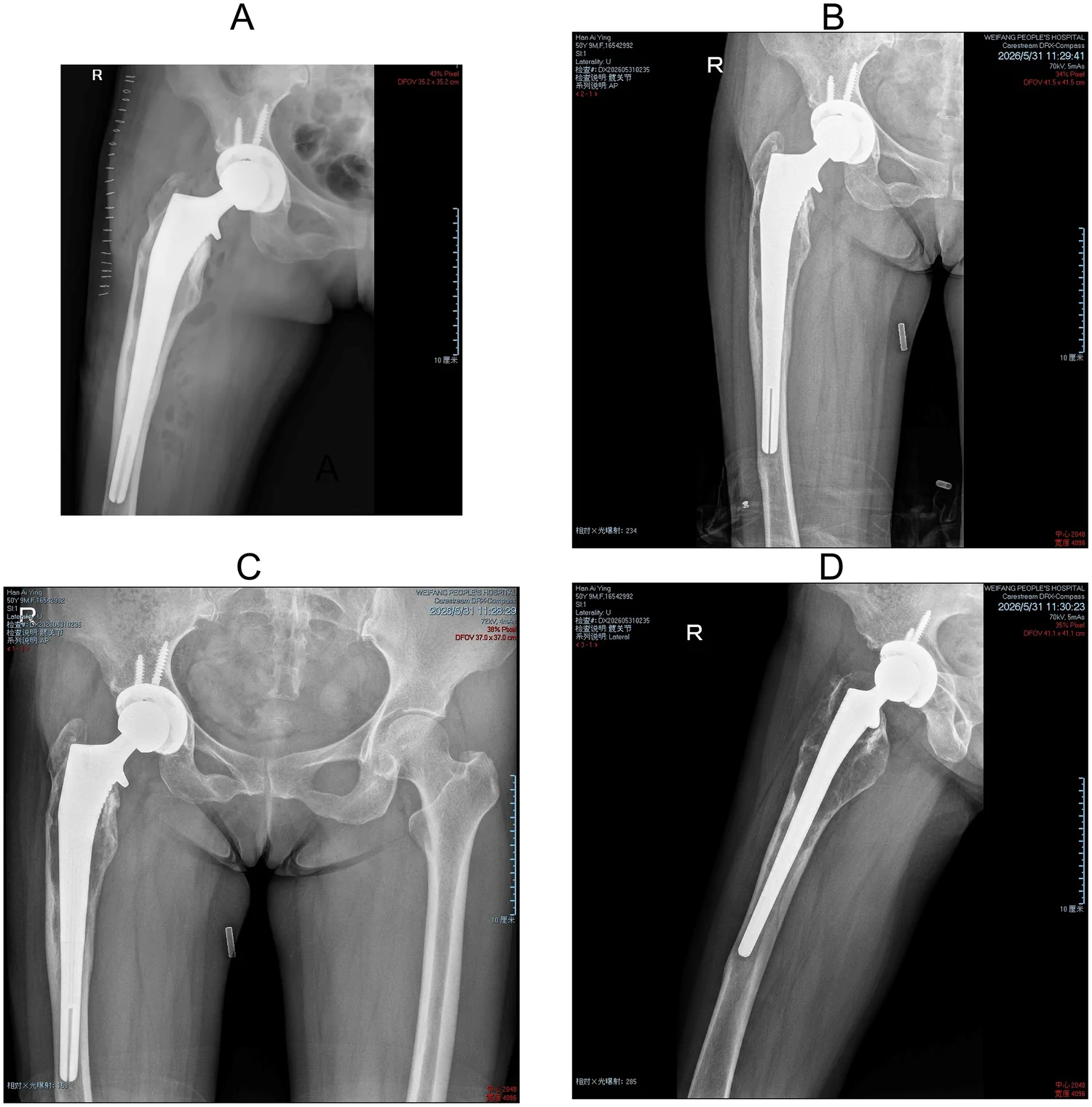
Postoperative and follow-up radiographs. **(A)** Anteroposterior radiograph of the right hip obtained immediately after surgery, showing appropriate alignment and fixation of the cementless revision stem and acetabular component. **(B–D)** Anteroposterior and lateral radiographs at 6-month follow-up, demonstrating stable implant fixation without evidence of subsidence, periprosthetic radiolucency, or loosening.

The patient's immediate postoperative course was unremarkable. Radiographs confirmed appropriate implant positioning (Figure 4A). Notably, the patient was able to ambulate with full weight-bearing using a walker on the first postoperative day. She demonstrated the ability to actively flex the hip beyond 90° independently and perform deep squatting maneuvers without difficulty, and reported no restrictions in toileting activities regardless of body position. She was discharged home on postoperative day 4, ambulating independently without a walker. Postoperative radiographic measurements confirmed restoration of leg length (within 2 mm of the contralateral side), a femoral offset of 38 mm (contralateral: 36 mm), and a stem alignment within 2° of neutral in both coronal and sagittal planes.

The postoperative rehabilitation protocol was structured in four phases. Phase 1 (POD 0–3): Full weight-bearing with a walker was initiated on postoperative day one. Quadriceps isometric exercises and ankle pumps were started immediately. No standard hip precautions, such as the abduction-neutral position mandated after posterior approaches, were enforced. Phase 2 (Week 1 – Week 6): Strengthening exercises (straight-leg raises, side-lying hip abduction, and standing hip extension) were performed in sets of 15–20 repetitions, 2–3 sessions per day, each lasting 30–40 min. Ambulation with a walker was maintained during the first 2 weeks, with heel-strike emphasis and even stride. The patient was transitioned to a single cane at 3–4 weeks as tolerated. No deliberate restriction of hip flexion was imposed during this period, unlike the <90° limitation typical after posterior approaches. Phase 3 (Week 6 – Month 3): The patient was weaned from ambulatory aids to independent walking. Resistance training with elastic bands, step training, and proprioceptive exercises were introduced. Gait training focused on correcting the Trendelenburg gait pattern secondary to gluteus medius weakness. Phase 4 (After Month 3): Return to normal daily activities and low-impact recreational sports was permitted.

At the 6-month follow-up visit, the patient reported minimal to no hip pain (VAS score 0 at rest, 0–1 with prolonged ambulation) and had resumed unrestricted daily activities (Figures 4B–D). She was able to walk more than 1 km without significant discomfort and could ascend and descend stairs reciprocally without the use of a handrail. Physical examination demonstrated a painless range of motion with flexion to 110°, abduction to 35°, and internal rotation to 25°. Radiographs demonstrated a well-fixed prosthesis with no evidence of subsidence, loosening, or periprosthetic radiolucent lines. The right inferior pubic ramus lesion remained stable in appearance. Repeat Harris Hip Score at this time was 90 points (pain subscore 40/44, function subscore 42/47), reflecting a substantial functional improvement compared with the preoperative score of 64.

### Timeline

The clinical timeline of the patient's episode of care is summarized in Table 1.

**Table 1:** Timeline of the episode of care.

Time point	Event
4 years prior	Onset of progressive right hip pain
6 months prior	Significant worsening of pain and mobility
2025-12-20 (Preoperative)	HHS 64; imaging confirmed FD with shepherd's crook deformity (NSA 110°)
2025-12-21 Day 0 (Surgery)	THA via DAA with cementless revision stem; no osteotomy; no bone graft; blood loss 200 mL
Postoperative Day 1	Full weight-bearing with walker; active hip flexion >90°
Postoperative Day 4	Discharged home; independent ambulation without walker
6 months postop	HHS 90; return to farm work; radiographs show stable fixation

## Discussion

Fibrous dysplasia involving the proximal femur frequently leads to secondary hip osteoarthritis, with a considerable proportion of patients ultimately requiring total hip arthroplasty (8, 9). Currently, the literature provides limited guidance regarding optimal implant selection and postoperative outcomes for this unique population. Surgical decision-making, encompassing femoral component choice and the decision to perform corrective osteotomy, is typically individualized based on the extent of bone involvement and severity of deformity. The seminal study by Sierra et al. (6) first revealed the formidable challenges of THA in FD: at a mean follow-up of 15.7 years, 7 of 12 hips required revision (58%), and 3 of 5 cementless stems were revised for early loosening, suggesting inadequate fixation with first-generation cementless designs. However, more recent studies employing modern implant designs have demonstrated substantially improved results. Yao et al. (10) reported on 12 patients treated with 3D-planned corrective osteotomy, long cementless stems, and impaction bone grafting, achieving no revisions at a mean follow-up of 47 months. Garceau et al. (7) utilized conical fluted stems with routine proximal femoral allografting (80% of cases) and similarly achieved a low revision rate (10%), albeit with a 90% transfusion rate. Recently, Hayama et al. (11) reported a case of an elderly patient treated with a cemented stem without osteotomy, emphasizing a less invasive strategy for frail, osteoporotic individuals. Moharrami et al. (12) demonstrated the feasibility of bilateral long-stem cementless THA in a young patient, while Kahn et al. (13) provided an alternative strategy of hip resurfacing when the femoral canal was not amenable to stem insertion.

The present case occupies a distinct position within this treatment spectrum: a middle-aged female with monostotic FD and a classic shepherd's crook deformity, treated with a primary THA using a cementless revision stem via a DAA, without corrective osteotomy or structural bone grafting. This strategy enabled immediate full weight-bearing on the first postoperative day and yielded a Harris Hip Score of 90 at six months. The deliberate surgical decision-making in this case offers practical insights for future surgical management of THA in FD patients. While the early outcome is encouraging, it must be emphasized that these are preliminary results from a single case, and no conclusions regarding the safety or efficacy of this strategy relative to established techniques can be drawn.

The medullary canal in FD patients is often filled with tough fibro-osseous tissue, obliterating normal anatomical landmarks and posing a high risk of cortical perforation during reaming (14). In the present case, the canal was completely occluded by “fish-flesh”-like tissue, necessitating meticulous curettage prior to sequential, fluoroscopically guided reaming. Yao et al. (10) emphasized that thorough lesion debridement is a prerequisite for achieving reliable cementless fixation, as residual pathological tissue impedes osseointegration. Garceau et al. (7) utilized an extended trochanteric osteotomy to enhance visualization and lesion clearance, but deformity correction was accomplished with a separate osteotomy. Given the monostotic nature of the lesion and the absence of significant cystic change in this case, adequate canal preparation was achievable through the standard DAA without the need for an extended osteotomy, thereby minimizing soft tissue injury.

For shepherd's crook deformity, corrective valgus osteotomy is widely advocated to restore neutral mechanical alignment and facilitate stem passage. Yao et al. (10) performed 3D-planned osteotomies in 7 of 12 cases, all of which healed reliably with a mean limb lengthening of 2.8 cm. Garceau et al. (7) similarly employed extended trochanteric osteotomy for deformity correction. In contrast, based on preoperative templating confirming a viable trajectory for a straight stem despite the 110° neck-shaft angle, we elected not to perform an osteotomy. The Corail revision stem used in this case, with its rectangular dual-tapered geometry and full hydroxyapatite coating, possesses unique advantages in FD femurs: (i) the rectangular cross-section provides rotational stability through vertical engagement at the diaphyseal isthmus, rather than relying on proximal metaphyseal press-fit (15); (ii) the dual-tapered design permits gradual axial seating, reducing hoop stresses and the risk of intraoperative fracture (16); and (iii) the 150 μm HA coating can promote osseointegration even in the presence of residual fibrous tissue (17).

Garceau et al. (7) reported the use of proximal femoral allograft in 80% of hips in their series, with a higher utilization rate in polyostotic disease (80%) than in monostotic disease (40%). Yao et al. (10) employed impaction bone grafting in all cases to augment the bone-implant interface. In the present case, the monostotic lesion was confined to the proximal femur, and the residual cortical shell, though thin, possessed acceptable structural integrity. Consequently, we opted against the use of structural or impaction bone grafting, relying instead on the distal fixation afforded by the revision stem. The absence of subsidence and a radiographically stable implant at six months suggests that routine grafting may not be essential for monostotic FD with adequate diaphyseal bone stock, consistent with Sierra et al.'s observation that monostotic disease carries a more favorable prognosis than polyostotic disease (6).

The DAA was utilized in this case to preserve the abductor mechanism and posterior capsule. The patient's ability to ambulate with full weight-bearing on the first postoperative day and to perform unrestricted hip flexion without dislocation precautions is likely attributable, at least in part, to the soft-tissue preservation afforded by the DAA. However, in the absence of a direct comparison with alternative approaches in FD patients, these observations should be regarded as hypothesis-generating rather than conclusive evidence of superiority. To our knowledge, this is the first report of the DAA in FD with shepherd's crook deformity.

FD lesions are notably hypervascular due to aberrant angiogenesis within the fibro-osseous matrix (18). Garceau et al. (7) reported a transfusion rate as high as 90%. In contrast, intraoperative blood loss in the present case was approximately 200 mL, and no intraoperative transfusion was required. This favourable haemostatic profile is likely attributable to several factors beyond surgical technique: the monostotic nature of the lesion, which is inherently less extensive and less vascular than polyostotic disease; the avoidance of corrective osteotomy, which reduces bleeding from cancellous bone surfaces; and the omission of proximal femoral structural allografting, a procedure associated with substantial blood loss in the Garceau et al. series where allograft was used in 80% of predominantly polyostotic cases. Despite this favourable outcome, preoperative blood product reservation, routine tranexamic acid administration, and a low threshold for intraoperative cell salvage are recommended for all FD-THA procedures.

Intraoperative periprosthetic fracture is the most feared acute complication during THA for FD (19). No fracture occurred in this case, likely attributable to the low-stress insertion mechanism characteristics of this stem design and the meticulous, fluoroscopically guided canal preparation. Nonetheless, the thin cortices in FD remain critically fragile, and surgeons should maintain a low threshold for prophylactic wire cerclage.

This study has several important limitations. First and most critically, the follow-up is only six months, it's an exceptionally short period for evaluating THA in dysplastic bone. Late complications including aseptic loosening, stress shielding-induced proximal bone resorption, and delayed periprosthetic fracture may manifest years after the index procedure and are of particular concern in FD patients with abnormal bone remodeling capacity (6). A prospective surveillance protocol with scheduled follow-up at 2, 5, and 10 years has been established. Second, as a single case report of monostotic FD, these findings are not generalizable to polyostotic disease or more severe deformities (neck-shaft angle <90°). Third, 3D preoperative planning was not utilized. Fourth, the patient selection considerations proposed herein are derived from a single case and should not be interpreted as validated clinical guidelines.

## Conclusion

This case demonstrates that for a middle-aged patient with monostotic fibrous dysplasia and a moderate shepherd's crook deformity (neck-shaft angle 110°), primary total hip arthroplasty using a cementless revision stem via the direct anterior approach, without corrective osteotomy or structural bone grafting, can achieve immediate full weight-bearing, excellent early functional recovery (HHS 90 at six months), and stable implant fixation. However, these are strictly preliminary early-term results from a single case. We propose preliminary selection considerations for this simplified strategy: monostotic FD, neck-shaft angle ≥110°, absence of excessive anterior bowing, and adequate diaphyseal bone stock (cortical thickness >5 mm). This paradigm expands the therapeutic armamentarium for FD-related hip osteoarthritis, offering a less invasive alternative to traditional osteotomy-based reconstructions in appropriately selected patients. The long-term safety and durability of this simplified strategy remain unproven. Extended radiographic and clinical follow-up and larger comparative studies are essential before this approach can be considered a reliable alternative to established osteotomy-based reconstructions.

## Patient perspective

The patient reported: “Before the operation, I could hardly walk across my yard because of the pain. Now, six months later, I am back to working on the farm and can walk over a kilometer without discomfort. I can squat and use the toilet without any help. The surgery has given me my life back.”

## Data Availability

The original contributions presented in the study are included in the article/Supplementary Material, further inquiries can be directed to the corresponding authors.
